# Proceedings of the Eighteenth Annual Meeting of the Illinois State Dental Society

**Published:** 1882-07

**Authors:** 


### PROCEEDINGS OF THE EIGHTEENTH ANNUAL MEETING OF THE ILLINOIS STATE DENTAL SOCIETY.

[Held at Quincy, May 10, 1882.]

The meeting was called to order, with a large number of members present, by the President, Dr. A. W. Harlan, of Chicago. Prayer was offered by the Rev. F. A. Thayer, of the First Congregationalist Church of Quincy. Mayor Deaderick then, in a brief speech, gave a hearty welcome to the visitors in the name of the people of the city, to which Dr. Harlan responded. The roll was then called. Dr. E. D. Swain, of Chicago, was elected a member of the Board of Examiners to fill the vacancy caused by the death of Dr. M. S. Dean. The President appointed a committee of three, Dr. G. V. Black, of Jacksonville, Dr. Cushing, of Chicago, and Dr. Kitchen, of Rockford, to report resolutions on the death of Dr. Dean.

After the annual reports of the several committees and officers were read, President Harlan delivered his annual address; which was listened to with interest, receiving many compliments from the members.

The Secretary read a voluntary paper from Dr. Barrett, of Buffalo, N. Y., who sent casts of a peculiar case in his practice. The discussion which followed this paper was participated in by many. The society then adjourned until 2 P. M.

After some preliminary business the regular order of the meeting of the afternoon was taken up.

Dr. A. W. Stevens, of Chicago, read an essay on "Mechanical Dentistry," a subject which he treated well.

The next paper was by Dr. L. P. Haskell, of Chicago, "Random Thoughts from the Laboratory." He first spoke of the importance of the subject "Mechanical Dentistry," and then gave some suggestions to the younger members of the profession as to what a well regulated laboratory should contain, and as to his own mode of working. He advised that temporary teeth should be inserted within twenty-four hours after the teeth were extracted, citing an instance in his own practice, where a party had six teeth extracted at 5 o'clock in the evening and at 7;30 P.M. of the same evening ate supper with a new set of teeth.

The two papers then came up for general discussion, methods of work in the laboratory and of taking impressions, occupying much time. Dr. Hurt suggested that teeth should be set according to the features of the person. He had one rule for the close-fisted, another for the liberal, one for the fleshy, one for the angular and one for those neither fat nor lean.

On motion, the rules were then suspended to hear a paper from Dr. W. N. Morrison, of St. Louis, on "Artificial Crowns." He gave the substance only of a paper he had prepared, illustrating his remarks with models. He showed what his first effort had been in 1862, following this with the improvements he had made since that time. This subject is one of considerable interest to the profession, and to Dr. Morrison is due great credit for the attention he has so long given this subject.

The meeting then adjourned to meet the next morning.

SECOND DAY'S SESSION.

With a still larger number of members in attendance; the meeting was called to order by the President. Business of a miscellaneous nature was first attended to, after which came a discussion upon artificial crowns, in which Drs. Brophy, Matteson, Gilmer, Talbot and Morrison participated.

The next paper in order was one on "Operative Dentistry," from Dr. K. B. Davis, of Springfield. This subject was treated in a thorough manner and received good attention.

Dr. Crouse, of Chicago, followed with a paper on the "Materials and Methods for Saving the Teeth."

The subject was then announced as open to discussion to all members and visitors. By request, Dr. Allport, of Chicago, addressed the Society, and his remarks were listened to with much interest and attention.

Remarks from Drs. Kulp and Sitherwood followed on the relative qualities of gold used in filling teeth. Dr. Brophy spoke of the use of oxy-phosphate of zinc, for fillings, when Dr. Crouse startled the company by objecting emphatically to the use of phosphate of zinc or any other of the zinc preparations, beside making vigorous defense of his manner of separating teeth.

Action on the reception of applicants for membership was delayed until the afternoon session. Adjourned.

Afternoon meeting was well attended. Among the applicants for membership was a lady, Mrs. Kate C. Moody, of Mendota, a graduate from the Dental Department of the University of Michigan. Mrs. Moody was the first woman to apply for membership, but she was cordially received.

Dr. Otoffy, of the committee appointed to present resolutions on the death of Dr. Hawxhurst, reported:

*Whereas*—Dr. D. G. Hawxhurst, A.B.M.D., D.D.S., of Battle Creek, Michigan, a corresponding member of this society, died at Paris, France, February 16, 1882, be it

*Resolved*—That in the death of Dr. Hawxhurst the Illinois

State Dental Society, the Michigan State Dental and Medical Societies and the dental profession of the United States in general loose a bright ornament, an honored and valued member, that the family and friends of the deceased sustain an irreparable loss.

*Resolved*—That this society humbly bow to the will of the Supreme Ruler, express their heartfelt sorrow and deep sympathy with the family and friends.

*Resolved*—That a copy of these resolutions be spread on the records and that a copy be given to the city press and the different dental journals for publication.

The resolutions were unanimously adopted.

Then followed the unanimous election to membership of the following applicants: Dr. Thomas B. Wheeler, of Chicago; Dr. E. M. Robbins, of Carthage; Dr. J. D. Moody, of Mendota; Mrs. Kate C. Moody, D.D.S.; Dr. J. R. Phillips, of Chicago.

The discussion of the paper of the morning on "Materials and Methods of Filling Teeth," by Dr. Crouse, was continued by Dr. Black, of Jacksonville, who spoke at some length on the use of non-cohesive gold for preserving teeth. Dr. Gardiner then took the floor and after asking many question of the different members as to their use of cohesive or non-cohesive gold, insisted that many dentists who claim to use one thing exclusively, really use another or both. His points, were well made, and liberally applauded. A long discussion followed. Dr. Reed urged the use of English, not American, amalgams as filling for the teeth.

Dr. Brophy took issue with Dr. Crouse on the use of chloride of zinc for capping exposed pulps. He pronounced the practice pernicious and said that it caused unnecessary pain to the patient.

Dr. Stevens, of Chicago, spoke of treatment of exposed pulp, saying he had been much interested in the success the different members had experienced, but had waited to hear of failures. He had never treated a pulp exposed so as to bleed but that it died—no matter what treatment he used. He strongly opposed the wedging of teeth as advocated by Dr. Crouse.

Dr. Noyes called attention to the fact that an exposed pulp which was treated one year ago was alive to-day.

Dr. Stevens asked him to wait five years and if the pulp was alive, then, he would give up.

After remarks from several others, the subject was passed and a paper was read by Dr. Brophy, of Chicago, on "Caries and Necrosis of the Maxillary Bones."

The paper was a very interesting one, and the history and treatment of ten or twelve cases in his own practice added much to its interest.

At the conclusion of the reading of this paper, it was announced that carriages were in waiting to take the members of the society for a drive through the city. This pleasure was afforded by the courtesy of the resident dentists of Quincy and of Messrs. Durant and Oehlman of the Quincy Dental Depot.

An evening session was held, at which Dr. G. V. Black read a paper on the subject, so-called, "Rigg's Disease." This was followed by a discussion by several of the members. Adjournment at 10 P. M.

Instead of a regular session of the society, the morning of the third day was given up to clinics. The local dentists furnished a few patients and members of the society completed the list. Drs. Davis, Taggert, and Dorn were among the operators, in filling. Drs. Patrick, of Bellville, and Mattison, of Chicago, demonstrated the construction of gold crowns. Dr. Allport gave a very interesting clinic in the filling of a tooth with soft foil.

During the forenoon Dr. L. P. Haskill, of Chicago, exhibited the furnishings of a model dental laboratory.

Afternoon session was called to order by the President at 2:30 P. M. After some announcements, Dr. Cushing, of the Committee on Resolutions, on the death of Dr. M. S. Dean, submitted a memorial, paying a high tribute to the character and worth of the deceased. It set forth that Dr. Dean was one of the founders of the society, and thirteen years ago was elected President at the meeting in Quincy. Dr. Dean was instrumental in making the State Society what it is to-day. He was a manly man, a safe counsellor, a true friend. In his death the State Society had suffered a great loss. As Chairman of this committee he recommended that a committee be appointed to prepare a sketch of the life of the deceased to be spread upon the records of the society. After many tributes from other members to the character of the deceased, the report of the committee was adopted by a rising vote.

Dr. Stevens then called attention to the manufacturers' combination, and the representatives of Ransom & Randolph, of Ohio, and S. S. White, of Philadelphia, were allowed to explain their position. A ballot was then taken to elect Dr. Wilson, of Aurora, to membership.

Then the paper of Dr. Brophy on "Caries and Necrosis of the Maxillary Bones," and that of Dr. Black on so-called "Rigg's Disease," both of which were read on Wednesday, came up for discussion.

Dr. J. D. Murphy, of Bushnell, then read a paper on "Indications for, and the best method of Extracting Teeth," giving his own method, and the one which seemed the best to him.

This paper was followed by one from Dr. Swain, of Chicago, on "Dental Education." This gentleman was in favor of not too much education, but just enough. Dentists should be educated for dentistry, and not for theology, surgery, law, and other things. He objected to the course of study in dental colleges to-day, as requiring too much time of the student, for subjects of no benefit to him in his profession. In the discussion that followed, Dr. Allport took issue with Dr. Swain, saying he believed in laying the foundations broad and deep; in commencing at the bottom and building up. Some were in favor of learning dentistry first, and then medicine. That was commencing at the top of the edifice and building downwards. Dentistry was a part of the medical profession. It was a specialty, the same as surgery. It was necessary for every dentist to have a medical education, and every dentist in the profession would admit that he felt the need of medical knowledge.

After remarks from others the Society adjourned until 8 P.M.

When the Society assembled in the evening a further discussion of the subject of "Dental Education" occurred, after which Dr. J. R. Patrick read a paper on "High Civilization not the Cause of Tooth Decay."

The morning session of the fourth day of the convention was given up to business matters—election of officers, etc.

On motion, the President appointed a committee of three to report a plan for the examination of skulls in the large museums of the country.

On motion, Dr. Patrick was invited to show to the Society his appliances for, and methods of regulating teeth, concerning which there was an animated discussion.

Decatur was selected as the place for the next meeting. The following officers were elected for the ensuing year:

President, Dr. E. C. Stone, Galesburg; Vice-President, Dr. John J. R. Patrick, Belleville; Secretary, Dr. E. C. Noyes, Chicago; Librarian, Dr. H. H. Townsend, Pontiac; Executive Committee, Dr. C. A. Kitchen, Rockford; Dr. A. S. Walz, Decatur; Dr. L. H. McCoy, Pana. Meeting adjourned until afternoon.

Afternoon session was called to order with President Stone in the chair.

After some preliminary business, the regular order was followed and a paper was read by Dr. S. S. Taylor, of Streator, on "Quinine; its use in Dentistry." Dr. Taylor told of the discovery and virtues of quinine; its effects on the system; its uses in malarial diseases, and how best to use it in dentistry.

The next paper was by Dr. J. Campbell, of Bloomington, on "Periodontitis; Cause and Treatment." Dr. Campbell being absent, the paper was read by Dr. Sitherwood.

Some general remarks followed the reading of these papers. The Society then adjourned to meet the second Tuesday in May, 1883.

